# Bisphosphonate conjugation enhances the bone-specificity of NELL-1-based systemic therapy for spaceflight-induced bone loss in mice

**DOI:** 10.1038/s41526-023-00319-7

**Published:** 2023-09-18

**Authors:** Pin Ha, Jin Hee Kwak, Yulong Zhang, Jiayu Shi, Luan Tran, Timothy Pan Liu, Hsin-Chuan Pan, Samantha Lee, Jong Kil Kim, Eric Chen, Yasaman Shirazi-Fard, Louis S. Stodieck, Andy Lin, Zhong Zheng, Stella Nuo Dong, Xinli Zhang, Benjamin M. Wu, Kang Ting, Chia Soo

**Affiliations:** 1 Division of Plastic and Reconstructive Surgery, Department of Surgery, David Geffen School of Medicine, University of California, Los Angeles, Los Angeles, CA 90095 USA; 2 Department of Orthopaedic Surgery and the Orthopaedic Hospital Research Center, David Geffen School of Medicine, University of California, Los Angeles, Los Angeles, CA 90095 USA; 3 Herman Ostrow School of Dentistry, University of Southern California, Los Angeles, CA 90089 USA; 4 Department of Bioengineering, Henry Samueli School of Engineering and Applied Science, University of California, Los Angeles, Los Angeles, CA 90095 USA; 5 Forsyth Institute, Cambridge, MA 02142 USA; 6 School of Dentistry, University of California, Los Angeles, Los Angeles, CA 90095 USA; 7 Space Biosciences Division, NASA Ames Research Center, Moffett Field, CA 94035 USA; 8 BioServe Space Technologies and Aerospace Engineering Sciences, University of Colorado, Boulder, CO 80303 USA; 9 Office of Advanced Research Computing, University of California, Los Angeles, Los Angeles, CA 90095 USA

**Keywords:** Medical research, Drug delivery

## Abstract

Microgravity-induced bone loss results in a 1% bone mineral density loss monthly and can be a mission critical factor in long-duration spaceflight. Biomolecular therapies with dual osteogenic and anti-resorptive functions are promising for treating extreme osteoporosis. We previously confirmed that NELL-like molecule-1 (NELL-1) is crucial for bone density maintenance. We further PEGylated NELL-1 (NELL-polyethylene glycol, or NELL-PEG) to increase systemic delivery half-life from 5.5 to 15.5 h. In this study, we used a bio-inert bisphosphonate (BP) moiety to chemically engineer NELL-PEG into BP-NELL-PEG and specifically target bone tissues. We found conjugation with BP improved hydroxyapatite (HA) binding and protein stability of NELL-PEG while preserving NELL-1’s osteogenicity in vitro. Furthermore, BP-NELL-PEG showed superior in vivo bone specificity without observable pathology in liver, spleen, lungs, brain, heart, muscles, or ovaries of mice. Finally, we tested BP-NELL-PEG through spaceflight exposure onboard the International Space Station (ISS) at maximal animal capacity (*n* = 40) in a long-term (9 week) osteoporosis therapeutic study and found that BP-NELL-PEG significantly increased bone formation in flight and ground control mice without obvious adverse health effects. Our results highlight BP-NELL-PEG as a promising therapeutic to mitigate extreme bone loss from long-duration microgravity exposure and musculoskeletal degeneration on Earth, especially when resistance training is not possible due to incapacity (e.g., bone fracture, stroke).

## Introduction

Planetary exploration by astronauts may be realized in the next decade. The National Aeronautics and Space Administration (NASA) has scheduled crewed missions to Mars in 2030, with even earlier 2024 plans for extended crewed missions to the Moon^1–3^. However, major spaceflight health risks, such as exposure to microgravity, high radiation levels, and systemic fluid shifts contribute to physiological deterioration, encompassing muscle atrophy, weakened bones, decreased immune function, altered vision, and cardiovascular disease^4,5^. In particular, decreased mechanical loading due to microgravity induces bone loss at a rate 12-times greater than on Earth^6,7^. Astronauts in low Earth orbit may experience bone loss up to 1% per month, endangering astronaut skeletal health and increasing risk for fragility fractures during long-duration spaceflight and later in life^6,8,9^.

NASA’s current mitigation strategy relies on exercise-induced mechanical loading^10,11^ to promote osteoblast and inhibit osteoclast functions as well as bolster bone formation^12–14^; however, persistently high urinary excretion of bone and collagen degradation markers in crewmembers during up to six months of microgravity exposure^15,16^ suggest this strategy is imperfect^17,18^. Furthermore, exercise is only accessible to uninjured crewmembers since vigorous mechanical loading may be contraindicated after bone fracture^19–21^ occurring during mission activities^22^. Collectively, astronauts currently spend a theoretical cumulative of 4.5 h weekly on resistive exercise tasks^23,24^, limiting available crew time and occupying valuable payload space with equipment^10^.

In theory, exercise supplemented with anti-osteoporotic medication provides reliable protection and could reduce exercise and equipment requirements. For example, astronauts engaged in only exercise-induced loading during 5.5 months of microgravity exposure exhibited significantly decreased bone density in femoral neck, trochanter, hip, pelvis, and lumbar spine regions, while supplementation with weekly oral administration of bisphosphonates (alendronate) slowed bone loss in these sites^25^. However, dyspepsia and abdominal pain are common side effects that cause patients to discontinue bisphosphonate therapy—and in one report, up to 20% of astronauts discontinued bisphosphonate therapy^25^. Thus, studies on potentially more optimal in-flight pharmacologic countermeasures to address the bone health risks of long-duration spaceflight are needed^18^.

Nel-like molecule-1 (NELL-1) is a secreted protein that induces significant in vivo bone formation in multiple small and large animal models^26–29^ including non-human primates^30^. Like mechanical loading^14,31,32^, NELL-1 upregulates osteoblast function, inhibits osteoclasts, and activates Wnt/β-catenin signaling pathways^33–35^ through its binding with Cntnap4 and integrin β1^34,36,37^. NELL-1 loss-of-function studies demonstrated protective function against osteoporosis as *Nell-1* haploinsufficient mice with reduced endogenous NELL-1 developed reduced bone mineral density (BMD), reduced osteoblast activity, increased osteoclast activity, and increased bone fragility with age^34^. Additionally, therapeutic use of recombinant human NELL-1 reverses osteoporotic phenotypes in several animal models. Local delivery of NELL-1 effectively recovers low mineral density in osteoporotic sheep spine and in an ovariectomized rat model^34,38^, while systemic treatment induced significant bone formation in either ovariectomized^34^ or non-ovariectomized^30^ mouse models. In humans, genome-wide analyses of 2,073 participants in the original Framingham osteoporosis study^39^ showed that rs10766761, located in intron 12 of the NELL-1 gene, was nominally associated with low BMD of both the femoral neck (*P* = 7.9 × 10^−4^) and the lumbar spine (*P* = 3.3 × 10^−4^)^40,41^. Additionally, NELL-1 reached a major clinical milestone when it received Australian Human Research Ethics Committee (HREC) approval in a multicenter pilot clinical trial to evaluate the safety and efficacy of NELL-1/DBX® in adult subjects with degenerative disc disease (DDD) undergoing spine fusion surgery.

In this observational study, we tested BP-NELL-PEG, the conjugation of alendronate with the large 1,195kD PEGylated recombinant human (rh)NELL-1, in a Center for the Advancement of Science in Space (CASIS) and NASA sponsored research project (Rodent Research 5 (RR-5)) on the International Space Station (ISS) to treat spaceflight-induced bone loss in mice. We previously introduced PEGylation of rhNELL-1 (NELL-PEG) and improved its half-life from 5.5 to 15.5 h, while increasing tissue distribution up to three-fold in femur, tibia, vertebrae, and calvarial tissues of mice^42^. Though this was a marked improvement over native (i.e., unPEGylated) NELL-1 protein, we found the highest concentrations of NELL-PEG 48 h after administration in the liver and spleen^42^. We sought to further improve bone uptake and reduce systemic distribution *via* bisphosphonate conjugation, which specifically targets molecules to bone^43^. In this study, we conjugated the bisphosphonate (BP) alendronate as a bio-inert drug carrier to NELL-PEG (BP-NELL-PEG), in order to enhance bone-specificity, enable intraperitoneal delivery, and reduce dosing frequency from every 7 days to every 14 days to meet the technical demands of this ISS study. Overall, we found that BP-NELL-PEG systemic therapy effectively reversed structural bone deterioration induced by long-duration spaceflight in mice.

Our 30-week-old BALB/c mouse osteoporosis model also helps address critical knowledge gaps in rodent skeletal changes under microgravity. We report longitudinal DXA and microCT bone data in spacefaring BALB/c mice – a rodent strain that, to our best knowledge, has never previously been studied in the context of spaceflight-induced bone loss^44^—despite its frequent usage on Earth for osteoporosis therapy testing and optimal osteoporotic response to ovariectomy, hypogonadism, and glucocorticoid administration compared to other strains^45,46^. Furthermore, the aged 30-week-old mice in this study provide valuable information about spaceflight adaptations in skeletally-mature animals, which exhibit less bone formation at baseline and demonstrate more drastic microgravity-induced bone loss than young, growing mice^47^. Given that prior spaceflight rodent studies have predominately evaluated young rodents^44^, these aged 30-week-old animals are particularly important for understanding bone loss in adult human astronauts.

## Results

### BP-NELL-PEG synthesis and characterization in vitro

**Fig. 1:**
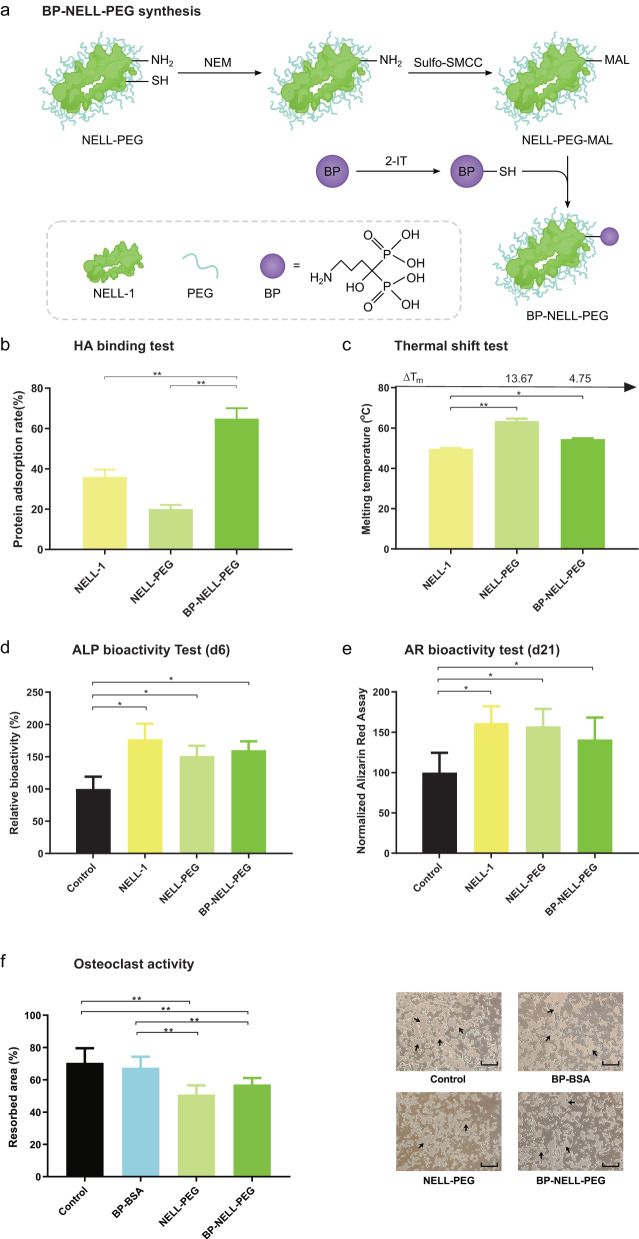
BP-NELL-PEG synthesis and characterization in vitro. **a** The chemical reaction illustration between BP (alendronate) with NELL-PEG. **b** The hydroxyapatite (HA) binding test showed that after BP conjugation, binding affinity of BP-NELL-PEG increased up to 3 times compared to NELL-PEG. **c** The thermal stability of BP-NELL-PEG was significantly increased compared to unmodified NELL-1. **d**, **e** Both ALP and AR bioactivity tests showed that the activity levels between NELL-1, NELL-PEG and BP-NELL-PEG were comparable and were all significantly higher than control group. **f** Von Kossa staining on the calcium phosphate-coated plates confirmed that BP-NELL-PEG inhibits OC bone resorptive activity in vitro. Compared to blank control, BP-BSA carrier control did not show a notable anti-OC effect, indicating that BP is bioinert. This indicates that BP-conjugation of NELL-PEG did not introduce additional anti-OC effect. Note: *p < 0.05, **p < 0.01 (two-tailed Student’s *t* test). HA hydroxyapatite, ALP alkaline phosphatase, AR alizarin red, BP bisphosphonate (alendronate), NELL-PEG PEGylated NELL-1, BP-NELL-PEG BP-conjugated NELL-PEG, NEM N-ethylmaleimide, MAL Maleimide, 2-IT 2-iminothiolane, Sulfo-SMCC Sulfosuccinimidyl-4-(N-maleimidomethyl)-1-cyclohexane carboxylate, BSA Bovine serum albumin.

To create a more stable, bone-targeting NELL-1 therapeutic, we first PEGylated NELL-1 (NELL-PEG) to improve its stability and then conjugated NELL-PEG with BP (BP-NELL-PEG) to enhance its ability to target bone (Fig. 1a). We selected alendronate, a nitrogen-containing BP, for conjugation with NELL-PEG because it has the highest binding affinity to bone among BPs that contain an amine group (-NH_2_), which can be used for conjugation with proteins^48^. The pharmacological mechanism of action of nitrogen-containing BP is by binding to and inhibiting the osteoclast (OC) enzyme farnesyl pyrophosphate synthase (FPPS), which in turn reduces post-translational prenylation of small GTPase proteins that are important in OC cell function and survival^49^. Furthermore, BP’s anti-OC function is structure-dependent, meaning that the specific orientation of the nitrogen atom in BP is responsible for the anti-osteoclastic pharmacological activity^49,50^. Nitrogen-containing BP’s anti-OC function can be inactivated by either removing nitrogen or changing the structural conformation of nitrogen in relation to the bisphosphonate group^51^. The latter method was used to inactivate BP’s anti-OC function in BP-NELL-PEG. BP conjugation with protein displaces the nitrogen away from the bisphosphonate group, affecting its binding to FPPS and reducing its anti-OC function to be practically ‘bioinert’, while retaining the bone binding ability.

We systematically characterized the physical chemistry of BP-NELL-PEG in vitro. First, fluorometric assay determination of the degree of BP conjugation revealed the NELL-1: BP: PEG molar ratio to be 1:3:19. Second, we conducted a hydroxyapatite (HA) binding affinity test to determine the bone targeting ability of BP-NELL-PEG. As shown in Fig. 1b, the HA binding affinity of BP-NELL-PEG at 64.6% was significantly greater than that of unmodified NELL-1 at 35.7% and of NELL-PEG at 19.8%. Third, thermal shift testing revealed that the thermal stability of BP-NELL-PEG was significantly higher than native NELL-1 (Fig. 1c) with the melting temperature of BP-NELL-PEG at 54.5 °C, and the shift amount (ΔT) at 4.75 °C relative to native NELL-1. Fourth, to assess whether PEGylation and BP conjugation would impair NELL-1 bioactivity, we examined the in vitro bioactivity of BP-NELL-PEG by alkaline phosphatase (ALP) assay and alizarin red (AR) mineralization assay. As shown in Fig. 1d, e, the ALP and AR bioactivities of BP-NELL-PEG were significantly enhanced compared to blank control, while there was no statistical difference among native NELL-1, NELL-PEG and BP-NELL-PEG treated groups. Importantly, the ALP and AR bioactivity assays demonstrated the chemical reactions involved in PEGylation and BP conjugation to structurally modify native NELL-1 did not impair its in vitro osteogenic effects. NELL-1’s large molecular mass (over 700 kDa) may confer an advantage over other smaller protein drugs (e.g., bone morphogenic protein BMP-2 with a molecular mass of 26 kDa) as it allows NELL-1 to withstand several surface modifications without notably affecting bioactivity^52^.

To explore BP-NELL-PEG effects on osteoclasts (OCs) and to verify that conjugation with NELL-PEG protein inactivates BP’s anti-OC function, we treated OCs cultured on calcium phosphate coated plates and quantified the resorbed area (Fig. 1f). We used BP-conjugated bovine serum albumin (BSA; hence, BP-BSA) as a carrier control group to examine for any anti-OC effect of BP (in the form of decreased calcium phosphate resorption). BP-BSA carrier control did not show any notable anti-OC effect compared to blank control, supporting the premise that the BP conjugate is bioinert in vitro. Also, both NELL-PEG and BP-NELL-PEG groups showed an expected strong anti-OC effect *via* decreased calcium phosphate coating resorption consistent with NELL-1’s known pro-osteoblast and anti-OC functions^34^, with no significant difference between them. Overall, we determined that BP-NELL-PEG displayed significantly increased HA affinity and thermal stability compared to NELL-PEG and native NELL-1, and that conjugation inactivated BP’s anti-OC effects without diminishing NELL-1’s osteogenicity.

### Biodistribution study in vivo

**Fig. 2:**
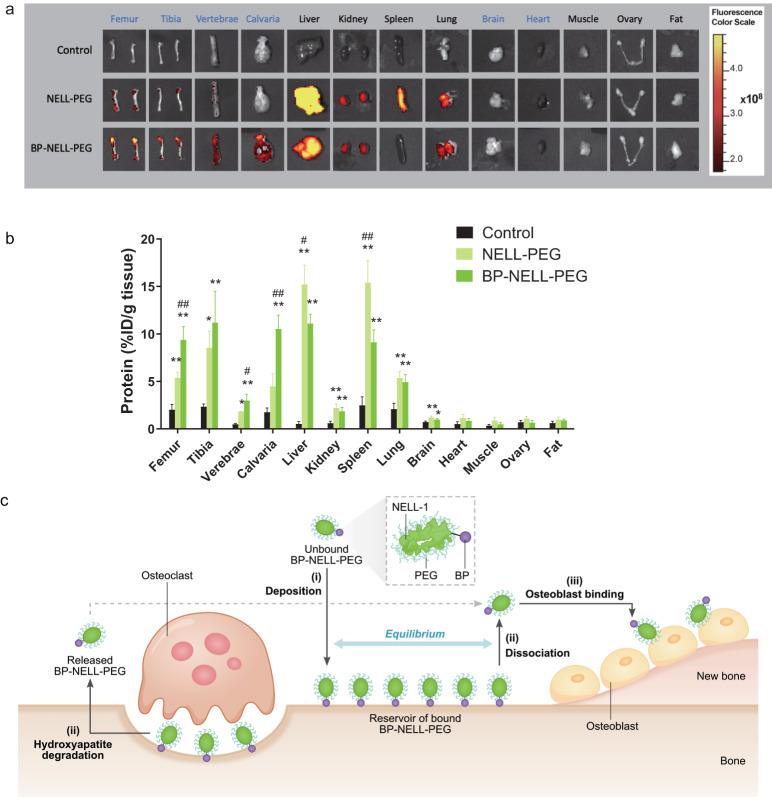
Biodistribution and BP-NELL-PEG’s mechanism of action. Study showed BP modification improved protein targeting to bone tissues in vivo. **a** Mice were intravenously injected with VivoTag680XL-labeled proteins, PBS/PEG injected mice were used as background control. The mouse organs were imaged by the IVIS Lumina II optical imaging system ex vivo at 48 h post-injection. Compared with the NELL-PEG group, BP-NELL-PEG showed a higher retention in bone tissues (e.g., femurs, vertebrae and calvaria), with reduced deposition in non-skeletal organs (e.g., liver and spleen). **b** Quantification of the protein deposited in different organs. The protein uptake in the brain, heart, muscle, ovary and fat are minimal. **c** Hypothetical model of BP-NELL-PEG’s mechanism of action. (i) Following systemic injection, BP-NELL-PEG travels to bone and deposits on hydroxyapatite (HA) in bone, then forms an equilibrium of the drug bound/unbound to HA primarily by electrostatic interaction between BP and HA. This creates a reservoir of BP-NELL-PEG on the bone surface. (ii) BP-NELL-PEG is released either by BP’s dissociation from HA or osteoclast-mediated HA degradation, and (iii) BP-NELL-PEG binds to the receptors of nearby osteoblasts and facilitates new bone formation. Note: NELL-PEG or BP-NELL-PEG vs control, *p < 0.05, **p < 0.01 (two-tailed Student’s *t* test); NELL-PEG vs BP-NELL-PEG, #*p* < 0.05, ##*p* < 0.01 (two-tailed Student’s *t* test).

We used fluorescent VivoTag 680XL tagged proteins to examine biodistribution and bone-targeting of BP-NELL-PEG compared with NELL-PEG following intravenous administration. We found the fluorescent signal intensity of BP-NELL-PEG in bone (femur, vertebrae, calvaria) to be significantly higher than that of NELL-PEG (Fig. 2a, b). Conversely, BP-NELL-PEG accumulation in the liver and spleen was significantly decreased by 27% and 41%, respectively, compared to NELL-PEG. Fluorescent signal intensity of BP-NELL-PEG was negligible in most non-skeletal organs (fat, ovary, muscle, heart, brain) except for a notable intensity in the liver, spleen, kidney and lung, likely attributed to the enhanced permeability and retention effect of these organs^53^. Deposition of NELL-PEG and BP-NELL-PEG in the brain was miniscule, because NELL-1 and its larger variants (NELL-PEG and BP-NELL-PEG, with molecular masses of 700 kDa or greater) are large biomolecular therapeutics with poor diffusivity across the blood-brain barrier due to their size and hydrophilicity^54^. The observed difference in brain between NELL-1 variants and the control group is largely because the brain is a microvessel and blood rich organ and because NELL-1 variants have longer residency time in blood^42^. Thus, even though NELL-1 variants have poor diffusivity across the blood brain barrier, they resided in brain microvessels and were detected.

### Osteogenicity study in ovariectomy-induced osteoporotic mice

Prior to the application of BP-NELL-PEG in our spaceflight study, we evaluated the osteogenic efficacy of BP-NELL-PEG in an ovariectomy (OVX)-induced osteoporotic mouse model. Previously, we found NELL-PEG (at 1.25 mg kg^−1^ NELL-1 dose) to effectively enhance bone volume and BMD when injected intravenously *via* mouse lateral tail vein every 7 days^42^. To meet the technical demands of the spaceflight study, our goal was to enable an easier intraperitoneal administration route (with a higher tolerated max injection volume than intravenous) and to reduce the frequency of injection from every 7 days to every 14 days. We also performed a preliminary BP-NELL-PEG dosing study using NELL-1 doses of 5 mg kg^−1^ and 10 mg kg^−1^ *via* intraperitoneal injection every 14 days, and we determined that 10 mg kg^−1^ formed more new bone without notable side effects (data not shown). Accordingly, for this study, we used the 10 mg kg^−1^ NELL-1 dose to compare the osteogenic effect of BP-NELL-PEG vs. NELL-PEG when injected every 14 days intraperitoneally in OVX mice. Importantly, we also tested a control group receiving BP-BSA to examine any anti-OC effects of the BP carrier that may contribute to increased BMD or percent bone volume (bone volume/tissue volume, BV/TV). MicroCT analysis was performed on the distal femur, proximal tibia and lumbar vertebrae. As shown in Supplementary Fig. 1, BP-NELL-PEG significantly improved trabecular BMD and BV/TV in all three bone sites to a level comparable to or higher than Sham control, while NELL-PEG without BP in the 14-day injection regimen only significantly increased BMD in the distal femur and BV/TV in the distal femur and proximal tibia to levels equal or greater than Sham control. Importantly, BP-BSA carrier control did not demonstrate any effects on BMD or BV/TV after 8 weeks of injection. Collectively, these data show that improved BMD and BV/TV is likely due to enhanced bone targeting by the “BP” component of BP-NELL-PEG, rather than any inherent anti-OC activity of conjugated BP.

### Osteogenicity study in spaceflight mice

**Fig. 3:**
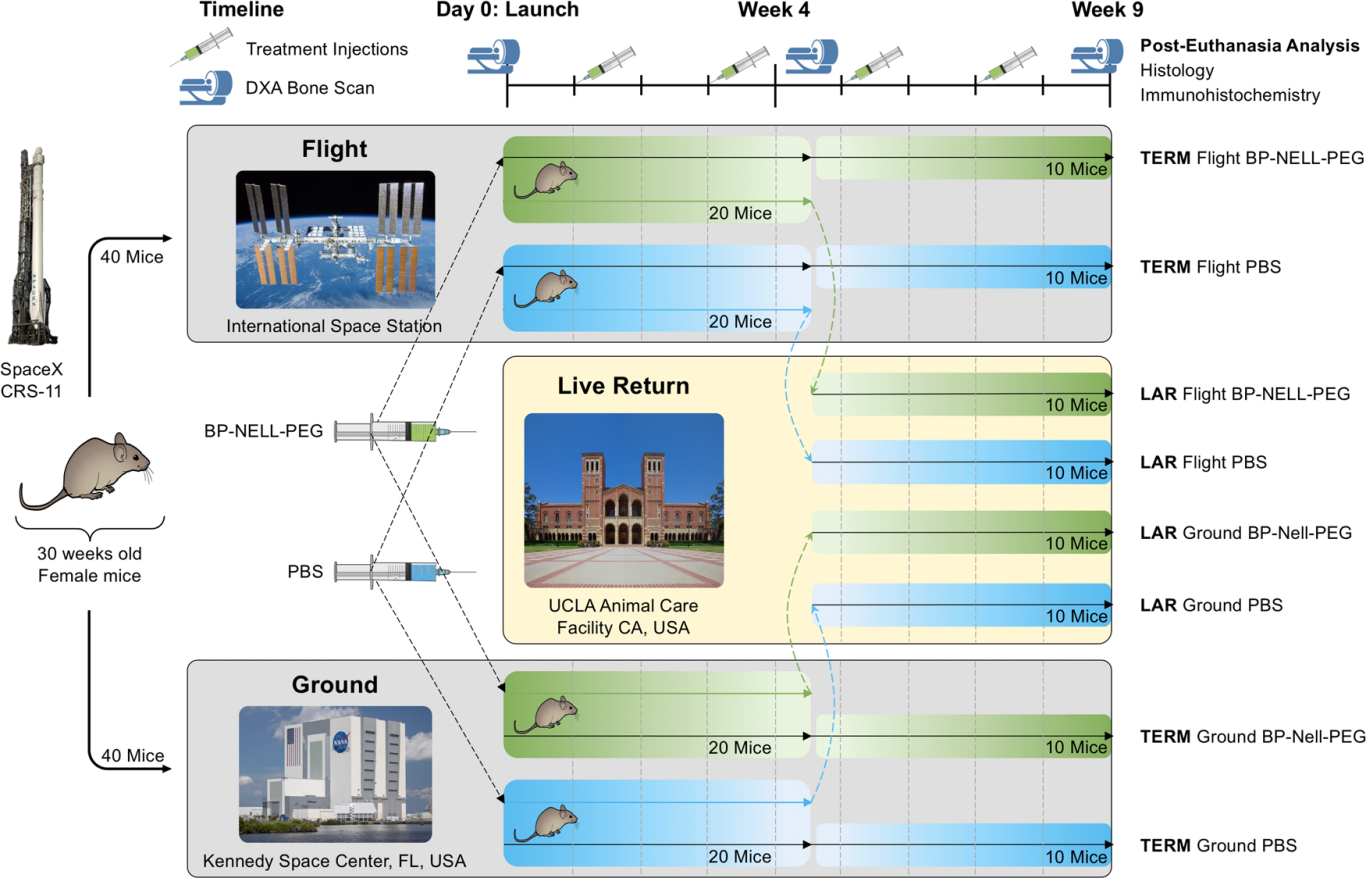
Experimental Design of RR-5 spaceflight mission. Mice were aged to 30 weeks at launch to ensure skeletal maturity. Forty flight group animals were flown *via* the SpaceX CRS-11 rocket and housed in the International Space Station (ISS). Forty matching ground control animals were housed in the Kennedy Space Center (KSC) in Florida. Starting at 1 week in orbit and approximately every 2 weeks thereafter, mice received either BP-NELL-PEG or PBS injections intraperitoneally. Halfway through the mission (at 4.5 weeks post-launch), half of the flight group animals (*n* = 20) were live-returned to Earth and transported to the animal care facility at UCLA to continue therapy for 4.5 additional weeks. Half of ground group mice (*n* = 20) in KSC were also transferred to UCLA for the same therapy. For all groups, DXA scans were performed at pre-launch, 4.5 weeks post-launch and 9 weeks post-launch. At 9 weeks post-launch, all animals were euthanized. Samples in the ISS and KSC were frozen and later returned to UCLA for dissection and analysis. The DXA bone scan icon is reproduced with permission from Shutterstock Inc., New York. Note: TERM ISS-housed until termination, LAR Live-Animal Return. SpaceX Falcon 9 rocket image cropped from original image "CRS-3" by Official SpaceX Photos at https://flickr.com/photos/130608600@N05/16234010894 [Creative Commons, Attribution 2.0 Generic (CC BY 2.0)]. Mouse illustration from original image "mouse profile" by Ann Kennedy at 10.5281/zenodo.3925921 [(Creative Commons, Attribution 4.0 International (CC BY 4.0)].

We next examined the therapeutic effect of BP-NELL-PEG (at 10 mg kg^−1^ NELL-1 dose every 2 weeks *via* intraperitoneal injection) in spaceflight mice. The experimental design for the spaceflight study is shown in Fig. 3 and described in Methods.

**Fig. 4:**
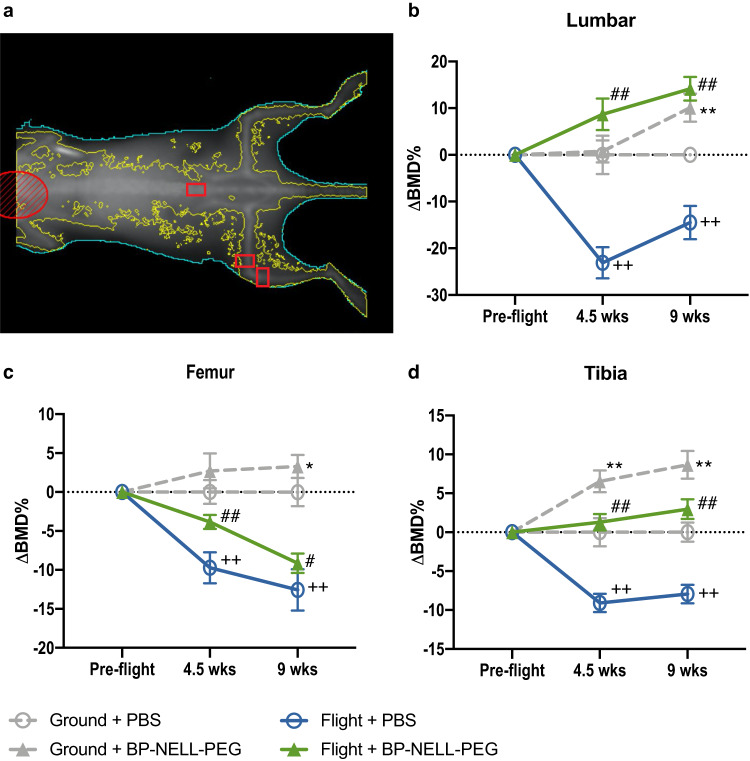
Longitudinal DXA bone densitometry of femur, tibia and lumbar vertebrae. DXA densitometry shows dynamic change of bone mineral density (BMD) from pre-flight through 9 weeks of spaceflight. **a** Mouse positioning and ROI locations in DXA (indicated by red boxes). **b** Relative BMD change in lumbar vertebrae. **c** Relative BMD change in distal femur. **d** Relative BMD change in proximal tibia. Values were normalized to ground + PBS group for each time point. Data are presented as means ± SEM. *n* = 10 per group. *p < 0.05, **p < 0.01 for comparison between ground + BP-NELL-PEG and ground + PBS. ^+^*p* < 0.05, ^++^*p* < 0.01 for comparison between flight + PBS and ground + PBS. ^#^*p* < 0.05, ^##^*p* < 0.01 for comparison between flight + BP-NELL-PEG and flight + PBS. *p*-values were calculated from a linear mixed model followed by likelihood ratio tests.

We performed dual-energy x-ray absorptiometry (DXA) bone densitometry to longitudinally monitor BMD changes in these mice at pre-launch, 4.5 weeks post-launch, and 9 weeks post-launch, utilizing a scan protocol set up in our previous study to achieve consistent mouse positioning and accurate region-of-interest (ROI) placement^55^ (Fig. 4a). Compared to the ground + phosphate-buffered saline (PBS) control group, the flight + PBS group showed significant decline of BMD in all bones (femur, tibia and lumbar) at 4.5 weeks and femur at 9 weeks. Meanwhile BP-NELL-PEG treatment groups showed a significantly increased BMD in all bones compared to control, in both ground and flight groups throughout the entire experiment (Fig. 4b–d).

**Fig. 5:**
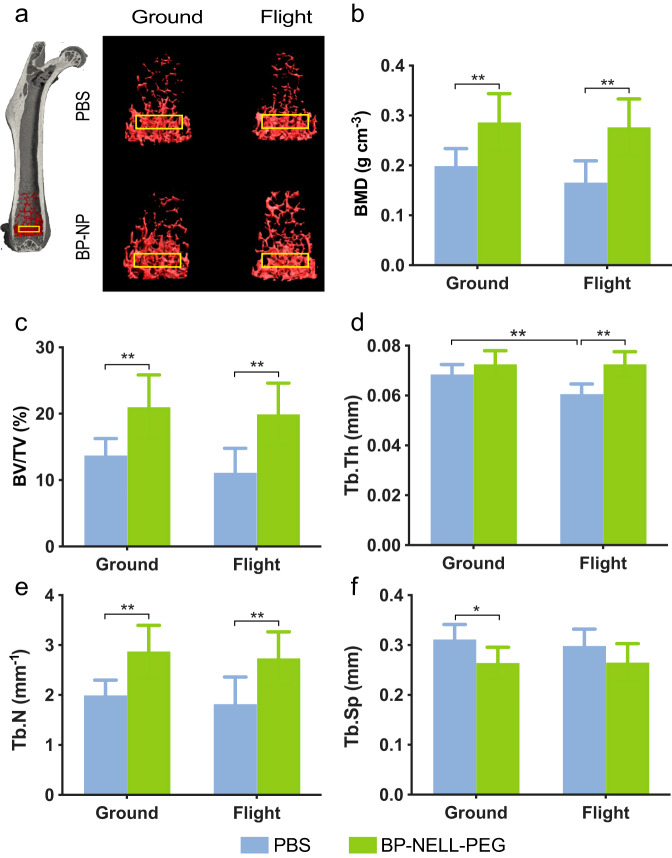
MicroCT analysis of femurs after 9 weeks of spaceflight. **a** Representative microCT 3D reconstruction images of the distal femoral metaphyseal region (ROI within the trabecular bone labeled in yellow boxes). **b**–**f** Histograms represent the trabecular structural parameters of the distal femur: bone mineral density (BMD), percent bone volume (BV/TV), trabecular thickness (Tb.Th), trabecular number (Tb.N), and trabecular separation (Tb.Sp). Data are presented as means ± SD. *n* = 10 per group. *p < 0.05, **p < 0.01 (One-way ANOVA with Tukey’s method as a post-hoc adjustment).

We then performed micro-computed tomography (microCT) scans on femurs, tibiae and lumbar vertebrae—common bones for evaluation of spaceflight-induced bone loss^17,47,56–60^—at the terminal time point (9 weeks post-flight) to examine trabecular bone. Specifically, we analyzed the distal metaphysis of femurs, proximal metaphysis of tibias, and caudal growth plate of L6 lumbar vertebral bodies, because these regions were previously characterized as particularly trabecular bone-rich^44,55^. As shown in Fig. 5, we found a spaceflight effect of decreased trabecular thickness in femurs of the flight + PBS group, compared to ground + PBS (Fig. 5d). Regarding the treatment effect, BP-NELL-PEG significantly improved trabecular BV/TV, BMD, and structural parameters in the analyzed region of femurs, in both ground and flight groups. Tibiae and lumbar vertebrae showed similar spaceflight and treatment effects (Supplementary Figs. 2 and 3). We also measured bone morphology of the distal femur cortical compartment and observed trending structural changes (*p* < 0.15, One-way ANOVA with Tukey’s post-hoc) in flight groups (Supplementary Fig. 4). These include decreased cortical area fraction (Ct.Ar/Tt.Ar, *p* = 0.108, One-way ANOVA with Tukey’s post-hoc), decreased cortical thickness (Ct.Th, *p* = 0.148, One-way ANOVA with Tukey’s post-hoc), and increased medullary area (Ma.Ar, *p* = 0.073, One-way ANOVA with Tukey’s post-hoc). Total cross sectional area was unchanged within the periosteal envelope (Tt.Ar). We did not detect any changes in cortical bone porosity (Ct.Po) with spaceflight exposure which is consistent with reports showing cortical porosity is unchanged in mice until ages much later than the 30-week-old animals used in this study^61^. Treatment with BP-NELL-PEG significantly increased both Ct.Ar/Tt.Ar and Ct.Th while significantly decreasing Ma.Ar in spaceflight mice and significantly increased both Ct.Ar/Tt.Ar and Ct.Th in ground control mice.

**Fig. 6:**
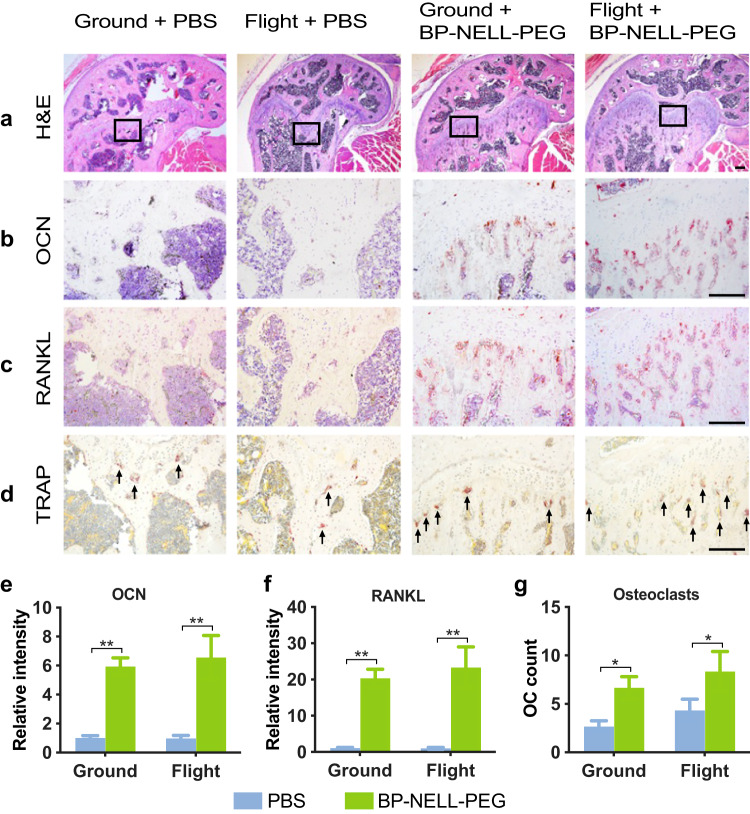
Histology, immunohistochemistry and TRAP staining of femurs. **a** Representative H&E images of distal femur shown at low magnification. **b** OCN and **c** RANKL immunohistochemistry representative images of distal femoral metaphyseal region shown at high magnification. The staining intensity of OCN and RANKL is quantified and summarized in graphs **e** and **f**, respectively. **d** Representative images of TRAP staining. Scale bar: 50 um. Multi-nucleic cells with positive TRAP staining (black arrows) identified as osteoclasts (OC). Quantification of OC counts are summarized in graph **g**. Data are presented as means ± SD. *n* = 3 per group. *p < 0.05, **p < 0.01 (two-tailed Student’s *t* test). Scale bar: 50um.

Histological analysis of femurs (Fig. 6) confirmed the treatment effect of BP-NELL-PEG, particularly at the distal metaphysis. BP-NELL-PEG treatment groups showed elevated bone formation, with a significant increase in osteocalcin (OCN) staining intensity as well as OC number and activity per receptor activator of nuclear factor kappa-Β ligand (RANKL) and TRAP staining in treatment groups. These findings demonstrate how BP-NELL-PEG treatment promotes overall bone formation and remodeling by activating both osteoblastic and osteoclastic activities in vivo.

## Discussion

In summary, conjugation of bisphosphonate (alendronate) to PEGylated NELL-1 improved overall drug stability, bone-specificity, and osteogenicity. We report that systemic therapy with BP-NELL-PEG mitigated spaceflight-induced bone loss in 30-week-old BALB/c mice during 9 weeks of microgravity exposure, characterized by improved BMD and BV/TV along with improved cancellous structure of the femur, tibia, and lumbar. We verified BP-NELL-PEG treatment’s effects on promoting bone growth and activating bone remodeling at the tissue level. We conclude that BP-NELL-PEG successfully reverses osteoporotic bone loss and is a viable pharmacologic countermeasure for use in spaceflight.

In this study, we bioengineered a BP-NELL-PEG drug molecule and tested its efficacy as a systemic therapy for osteoporotic bone loss. We found from our in vitro evaluation that bisphosphonate conjugation significantly improved hydroxyapatite binding and thermal stability, suggesting BP-NELL-PEG would demonstrate enhanced affinity for bone and resistance to heat mediated degradation in vivo. Importantly, we determined that conjugation inactivated BP’s anti-osteoclastic effect^62^ without diminishing NELL-1’s osteogenicity. Our in vivo evaluations corroborated our in vitro findings: BP-NELL-PEG significantly targeted to hindlimb, vertebral, and calvarial bone, while treatment with a bisphosphonate conjugate control (BP-BSA) did not alter bone mineral density (BMD) or percent bone volume (BV/TV), thus the BP moiety’s anti-osteoclastic effect was inactivated in BP-NELL-PEG. Bisphosphonate conjugation of NELL-PEG not only improved delivery to bone tissue up to two-fold, but significantly decreased off-target delivery to the liver and spleen by 27% and 41%, respectively, suggesting superior bone specificity. Based on the literature and our biodistribution findings, a hypothetical model of BP-NELL-PEG’s mechanism of action was developed (Fig. 2c). First, a reservoir of BP-NELL-PEG is formed on remodeling bone surface *via* BP’s high bone-affinity as a pyrophosphate analog – binding to hydroxyapatite by electrostatic interaction with BP’s phosphonate groups. As the binding between BP and hydroxyapatite is reversible with a dissociation constant (Kd) of 72 µM by Scatchard analysis^63^, BP-NELL-PEG can be slowly released from the bone surface and bind to nearby osteoblast (OB) receptors, integrin ß1^64^ and Cntnap4^65^, promoting bone formation. The osteoclast (OC)-mediated degradation of hydroxyapatite could also facilitate the release of BP-NELL-PEG from bone. Some BP-NELL-PEG molecules are thought to be ingested by OC while others are degraded by lysosomes.

Consistent with expectations^47,57,66^ DXA-assessed BMD in flight animals significantly decreased within the first 4.5 weeks of spaceflight and remained significantly below ground control values at the terminal 9-week timepoint. In agreement with Lee et al.^67^, our data also showed that bone loss did not progress at a constant rate during spaceflight, with BMD loss slowing or even reversing after the first 4.5 weeks after launch **(**Fig. 4). This trend in rodents is further supported by similar human astronaut bone loss data from long-duration missions^68^, and may reflect mammalian skeletal adaptations to long-term microgravity exposure. Of note, prior studies have also reported age-dependent variations in rodent response and adaptation to microgravity^47^. To our best knowledge, there are no prior reports on longitudinal BMD change in older (>30 weeks) mice exposed to longer than 1 month of microgravity. To further define the effects of long-term microgravity and age on bone, a new and ongoing NASA-supported study (RR-8) is investigating groups of young (10–16 weeks old) and older (30–52 weeks old) mice exposed to varying spaceflight durations (~30 and 60 days) on the ISS^69^.

Next, we showed BP-NELL-PEG systemic therapy significantly increased BMD and BV/TV in both ground and flight groups, with DXA and microCT evaluation demonstrating robust treatment effects. Treatment with BP-NELL-PEG during spaceflight exposure abated bone loss and we detected significantly enhanced BMD by DXA and significantly enhanced BMD and BV/TV by microCT. Additionally, BP-NELL-PEG treatment enhanced microCT-assessed cancellous structure of ground and flight animals by increasing overall trabeculae number or trabecular thickness in femur, tibia, and lumbar regions. This suggests BP-NELL-PEG not only improved bone formation, but also trabecular bone quality^70–73^, important for minimizing fracture risk in astronauts^74^.

Meanwhile, unlike our DXA data, microCT-measured BMD and BV/TV did not show significant differences between untreated ground and flight controls. We believe this discordance can be explained by differences in animal age and scanning methodology^75,76^. Coulombe et al.^47^. demonstrated differing bone microCT results between young and mature animals with up to 1 month of microgravity exposure. When comparing ground control and spaceflight groups, changes to trabecular microarchitecture and BMD were more readily detected in young (9-week-old) mice, which demonstrated significant changes to trabecular parameters of bone volume fraction (BV/TV), thickness (Tb.Th), spacing (Tb.Sp), and volumetric bone mineral density (vBMD) in both the proximal tibia and distal femur sites. In contrast, the same evaluation conducted in older (32-week-old) spaceflight mice revealed that only Tb.Th of the proximal tibia and vBMD of the distal femur were significantly changed by spaceflight. Here, trabecular vBMD of the proximal tibia was discordant between young and mature groups and between different trabecular sites in mature animals. This highlights the fact that bone remodeling in spaceflight is not only highly site specific, but also highly age specific.

With respect to methodology, DXA differs from microCT in several ways. DXA requires superimposition of cortical and trabecular compartments whose contributions are combined in the final measured outcome. This contrasts with microCT, where cortical and trabecular compartments are analyzed separately. Also, the limited analysis area of microCT necessitates small, carefully selected ROIs for each bone compartment. This is because mineral density changes occur heterogeneously in both trabecular^77^ and cortical^78^ compartments and nonrepresentative placement and/or insufficient number of ROIs assessed will inaccurately capture changes. Since cortical bone can contribute up to 90% of mineral loss in spaceflight^79^, its exclusion in trabecular microCT BMD measurements could explain the discrepancy between our aBMD and vBMD results. Furthermore, the differing methodologies could also explain why the predictive capabilities of cortical bone QCT evaluation are optimal only when combined with trabecular QCT parameters^80,81^ and even then, do not outperform DXA’s predictive capabilities.

Microgravity exposure disrupts bone homeostasis by uncoupling the tightly-regulated physiologic balance between osteoblastic bone formation and osteoclastic bone resorption^82–86^ in favor of excessive bone loss^9,17,18,47,57^. Specifically, studies show unloading inhibits osteoblast differentiation potential, downregulating gene expression of osteogenic markers alkaline phosphatase (ALP)^87^, runt-related transcription factor 2 (RUNX2), osterix, osteocalcin, type I collagen (Col I) and bone morphogenetic protein (BMP)^13^, and accumulates undifferentiated mesenchymal progenitors in bone marrow^88^. This inhibition impedes osteoblast activity, corroborated by reduced osteoblast presence^44^ and reduced serum bone formation markers, osteocalcin and alkaline phosphatase^57^ in spaceflight mice. Meanwhile, microgravity exposure simultaneously promotes osteoclastogenesis and bone resorption, evidenced by increased osteoclast presence^89^ and elevation of the TRACP-5b bone resorption marker^57^. This data along with observed bone loss^47,60,88,89^ suggest that microgravity-induced bone loss results from both osteoblast inhibition and osteoclast promotion, decreasing bone forming potential while increasing bone resorption. Interestingly, histological analysis on flight bone samples in the current study did not show a significant decrease in osteogenic marker (i.e., OCN) nor a significant increase in osteoclastic markers (i.e., TRAP, RANKL) (Fig. 6). Because our study has a longer spaceflight duration—9 weeks vs. 39 days—and used animals older (>30 weeks) than any prior rodent spaceflight bone studies^44^, we hypothesize that the lack of change in bone formation or resorption markers may result from skeletal adaptations of mature mice to long-term microgravity exposure, consistent with our microCT and DXA findings. Our osteoclast findings also align partially with a recent systemic review and meta-analysis, which showed that spaceflight-induced changes in osteoclast activity are inconsistent^44^. Although Fu et al. also reported significant decreases in osteogenic parameters (i.e., percentage of bone surface covered by osteoid and osteoid thickness), their study durations and animal ages were all less than 39 days and 20 weeks respectively, highlighting the need for the in-progress RR-8^69^ and similar future studies.

Our immunohistochemistry results verified BP-NELL-PEG treatment’s effects on promoting bone growth by showing increased trabecular bone formation and significantly increased staining intensity of osteoblast markers, osteocalcin and RANKL in the femur metaphysis in both ground and flight treatment samples, while more TRAP positive osteoclasts were simultaneously observed along the growth plate. Together, this implies BP-NELL-PEG treatment can promote bone turnover in favor of overall bone formation. Preservation of both osteoblast and osteoclast functions is significant because it suggests bone remodeling remains active. In contrast, anti-resorptive therapy, such as with bisphosphonates^25^ halt bone turnover, reducing bone quality and increasing fracture risk^10,90^.

Potential study limitations include firstly, that DXA superimposes cortical and trabecular compartments which are combined in the final measured BMD. This contrasts with microCT where cortical and trabecular compartments are analyzed separately, usually at differing bone sites^91^. Since cortical bone can contribute up to 90% of mineral loss in spaceflight^79^, its exclusion in our trabecular microCT analyses suggests that only limited comparisons can be made between our DXA and microCT data. Second, ISS research capacity is drastically limited in physical space and available crew time, making microCT, urine collection, and many other procedures unfeasible. For example, crew time limitations precluded detailed behavioral observation of research animals. Therefore, the effect of stress or other negative stimuli that could potentially affect bone loss during spaceflight, remains unclear. These experiments are also infeasible to repeat or expand upon under current ISS conditions, and limits our study to be observational in nature. Further data from future studies will help expound the mechanisms by which BP-NELL-PEG affects different bone cells (osteoblasts and osteoclasts) to achieve net bone formation. Third, the enrichment hut may have introduced huddling and bone loading that partially mitigated microgravity-induced bone loss. This is evidenced by our data showing less significant bone loss compared to earlier spaceflight studies^47^, as well as other enrichment hut studies that report the absence of expected significant microgravity-induced muscle loss^92^. Further studies on the enrichment hut are needed to evaluate its potential mitigative effect on microgravity-induced bone loss. Histologically, we found that BP-NELL-PEG activated OCs at the femur metaphysis by showing elevated RANKL and TRAP staining (Fig. 6). To minimize BP’s anti-OC function, we inactivated BP by changing the structural conformation of BP’s nitrogen atom during conjugation per established protocols^50,51^ and then further verified pharmacologic inactivation of BP in vitro (Fig. 1f). Nevertheless, this study lacks direct in vivo evidence of BP’s inactivation, and a BP-BSA carrier control group should be included in future rodent studies.

In summary, spaceflight exposure promotes bone fragility phenotypes^5,17,22,93,94^ which compromise astronaut skeletal health and contribute to increased risk of fracture. Recent efforts to investigate pharmacological countermeasures for spaceflight-induced bone loss yielded promising results; however, the adverse effects make some therapeutics unsuitable for use in long-duration missions. For example, subcutaneous injection of osteoprotegerin^57^, although able to diminish spaceflight-induced bone loss in the tibia and lumbar spine, obliterated osteoblast and osteoclast presence along bone surfaces. Likewise, oral alendronate supplementation along with exercise^25^ reduced bone loss in the femoral neck, trochanter, hip, pelvis, and lumbar spine, but induced dyspepsia and abdominal pain. Also, bisphosphonate use is associated with halted bone turnover and reduced bone quality, leading to brittleness, and increased mean bone age^10,90^. To develop a superior pharmacologic countermeasure, we bioengineered BP-NELL-PEG to improve the overall stability, specificity, and osteogenicity of PEGylated NELL-1 and enable its use in a spaceflight study on the technically intensive RR-5 spaceflight mission, as a potential systemic therapy for spaceflight-induced bone loss. Spaceflight-induced bone loss is correlated with disrupted osteoblastogenesis^88^, increased osteoclast presence^89^, and increased osteoclastic resorption^17^. These effects may be mediated through the Wnt/β-catenin pathway, since expression of Sclerostin, an inhibitor of osteoblast-mediated bone formation *via* the Wnt/β-catenin signaling pathway, is upregulated in response to microgravity exposure^95^. Therefore, BP-NELL-PEG, is particularly suited to counteract these underlying cellular changes because NELL-1 increases the expression and nuclear translocation of β-catenin in osteoblasts and osteoclasts, increasing osteoblast differentiation and inhibiting osteoclast-directed bone resorption^34^. NELL-1 also selectively promotes mesenchymal stem cells to undergo osteogenic differentiation^96^, inducing high expression of late-stage osteoblastic markers, such as osteopontin and osteocalcin, as well as mineralization^97^. Overall, BP-NELL-PEG systemic treatment successfully diminished spaceflight-induced bone loss without adverse effects on bone cell populations or bone turnover, posing distinct advantages for spaceflight. Additionally, although adipogenesis was not a focus of this study, we discovered, based on morphometric histological analysis, that BP-NELL-PEG treatment of spaceflight animals significantly reduced the number of marrow adipocytes in the femur, thereby reversing signs of spaceflight-induced bone aging^5,17,22,93,94^. Lastly, we contributed to the basic understanding of microgravity-induced bone adaptations in skeletally-mature rodents^98,99^ as well as the understudied BALB/c strain, by reporting longitudinal DXA-measured BMD and microCT-measured bone microarchitectural data in aged 30-week-old BALB/c mice. Altogether, we conclude that BP-NELL-PEG systemic treatment effectively prevents microgravity-induced bone loss in spaceflight mice. The results of this study highlight the future promise of BP-NELL-PEG treatment for preventing premature bone aging and fracture risk in astronauts during long-duration spaceflight.

## Methods

### BP-NELL-PEG synthesis

NELL-1 was first PEGylated according to our previous method^100^. Next, NELL-PEG was conjugated with BP through the following three steps: (1) NELL-PEG (5 mg/ml, 100 mM PBS, pH 7.0) was reacted with N-ethylmaleimide (NEM) solution (5 mM) for 30 min at 4 °C, and then reacted with Sulfosuccinimidyl-4-(N-maleimidomethyl)-1-cyclohexane carboxylate (sulfo-SMCC, 10 mM). The resulting solution was passed through a sephadex column, and then dialyzed against double-distilled water for 24 h at 4 °C to remove the unreacted small chemicals. The solution was then centrifugal filtered (molecular weight cut-off of 100 kDa). (2) Alendronate sodium (BP) was treated with 5 M HCl solution and purified by 95% ethanol, before drying the BP powder for 24 h. The obtained BP (20 mM in 100 mM PBS, pH 7.0) was thiolated with an equal volume of 2-iminothiolane (IT, 10 mM in 100 mM PBS, pH 7.0) for 2 h. (3) The resulting BP-IT was then directly added to the NELL-PEG solution in step (1) and reacted for 3 h at 4 °C. The resulting solution was passed through a Sephadex column to remove unreacted chemicals, and then dialyzed against double-distilled water for 24 h at 4 °C. The solution was centrifugal filtered (molecular weight cut-off of 100 kDa) and then lyophilized to obtain the final product, BP-NELL-PEG (frozen for 24 h and dried for 48 h). The lyophilized protein was reconstituted with PBS (pH 7.4) prior to use.

### BP-NELL-PEG characterization in vitro

We characterized BP-NELL-PEG in vitro by conjugation degree, HA binding affinity, thermal stability, osteogenic bioactivity and anti-OC activity.

1. We determined the conjugation degrees of PEG and BP in our synthesized BP-NELL-PEG using fluorometric assay as previously described^100^. Briefly, 36 µl of BP-NELL-PEG (1.0 mg/ml, PBS, pH 8.0) were mixed with 12 µl of fluorescamine solution (1.0 mg/mL) and incubated for 15 minutes at 25 °C in a 96-well microplate. NELL-1 and NELL-PEG were used as controls. The fluorescence intensity of the solution was determined by a plate reader (Infinite F200, Tecan Group Ltd.). The excitation wavelength was 390 nm and emission wavelength was 475 nm. The conjugation degree was calculated based on the modified amine amount over free amine amount in the protein.
2. We determined the HA binding affinity of BP-NELL-PEG using HA microparticles (5 ± 1 µm d50, NanoXIM). BP-NELL-PEG, NELL-PEG and NELL-1 were labeled with fluorescein isothiocyanate (FITC), added to HA solution, and mixed for 8 h. The supernatant was collected and the concentration of the protein in supernatant was determined by a fluorescent spectrophotometer at an excitation wavelength of 485 nm and an emission wavelength of 535 nm. The HA binding affinity of the protein was calculated using the equation below:
  1 $${\rm{HA\; affinity}}=\frac{{\rm{Protein\; added}}-{\rm{Protein\; detected\; in\; supernatant}}}{{\rm{Protein\; added}}}\times 100 \%$$
3. We determined the thermal stability of BP-NELL-PEG by thermal shift assay using a fluorescent dye, Sypro Orange, as previously described^100^. Five µl of protein (BP-NELL-PEG, NELL-PEG or NELL-1) (1.0 mg/ml), 2.5 µl of SYPRO orange (40x dilution) and 17.5 µl of PBS buffer (0.01 M, pH 7.4) were mixed in a 96-well microplate. The microplate was sealed and centrifuged for 2 minutes at2500 x*g*, and then placed into a 7300 real-time PCR system (Applied Biosystems, CA). The samples were heated from 298 to 368 K at a rate of 1 K/min, and the fluorescence intensity was monitored using SYBR as a reporter. The fluorescent signal of the samples was analyzed using a Boltzmann model and the melting temperature (T_m_) was obtained.
4. We evaluated the osteogenic bioactivity of BP-NELL-PEG in MC3T3 cells (Subclone 4, ATCC, CRL-2593) using alkaline phosphatase (ALP) assay and alizarin red (AR) mineralization assay. (i) ALP, a biomarker for early osteoblast differentiation, was used to determine the bioactivity of BP-NELL-PEG in MC3T3 cells. BP-NELL-PEG protein was added to the culture medium of MC3T3 and incubated for 7 days. The cells were then lysed by adding 200 µl lysis buffer (0.2% NP40 plus 1.0 mM magnesium chloride). The cells were scraped and centrifuged at 7500 × *g* for 5 min, and then 15 µl of supernatant were used to react with 200 µl of ALP substrate buffer under 37 °C for 15 minutes. The ALP signal was measured at 405 nm, and the data was normalized by protein content in corresponding wells. NELL-1 and NELL-PEG were tested using the same procedure. Measurements were performed 3 times for each sample. (ii) The ability of BP-NELL-PEG to facilitate inorganic calcium deposition during MC3T3 differentiation in vitro was determined by AR mineralization assay, with mineralization serving as a marker for late-stage osteogenic differentiation. BP-NELL-PEG protein was added into MC3T3 cell culture medium and incubated for 21 days in a 24-well plate. The culture medium was changed every 3 days. After calcium crystals formed at 21 days, the cells in wells were fixed with 10% formaldehyde and stained with alizarin red S solution (ARS; 40 mM, pH 4.2, 1 ml per well). After removing the excess ARS dye by rinsing 4 times with double-distilled water, the stained cells were subjected to visual inspection. Subsequently, the stained cells were scraped from the plate and the ARS in cells was dissolved by 400 µl of 10% acetic acid. The cell lysate was centrifuged at 8000 × *g* for 15 min. The supernatant was collected and pH value was adjusted to 4.3 by 10% ammonium hydroxide. The ARS in each well was then quantified at OD 405 nm. NELL-1 and NELL-PEG were tested using the same procedure. The measurements were performed 3 times for each sample.
5. We utilized in vitro OC culture to evaluate the anti-OC activity of BP-NELL-PEG and NELL-PEG, as well as confirm the pharmacologic inactivation of conjugated bisphosphonate using BP-conjugated bovine serum albumin (BP-BSA) carrier. First, OCs were isolated and cultured per methods described by Tevlin et al.^101^. Briefly, bone marrow was flushed from mouse femurs and the remaining bone tissue was gently crushed in flow cytometry buffer using a pestle and mortar. Bone marrow flush was centrifuged at 200 × *g* for 5 min at 4 °C to yield a cell pellet, which was then suspended and layered onto commercially available density gradient cell separation media. Isolated cells were seeded on 24-well plate (2 × 10^5^ per well) and cultured in macrophage stimulating media (PGE2 at 10^−7 ^M, M-CSF at 10 ng ml^−1^) for 3 days at 37 °C. After the macrophage induction stage, the cells were cultured continuously in OC induction medium (PGE2 at 10^−7 ^M, M-CSF at 10 ng ml^−1^, and RANKL at 10 ng ml^−1^) for 5 days until large, multinucleated OCs formed on the culture plate. Then NELL-PEG and BP-NELL-PEG were added to culture at 800 ng ml^−1^ of NELL-1 protein content, and BP-BSA was added at 408.8 ng ml^−1^ to ensure the same molar concentration of BP as BP-NELL-PEG. Von Kossa staining was performed on Day 3 post-treatment, using the previously reported standard method^101^ to visualize the un-resorbed calcium phosphate coating.

### Animals

We chose the age, gender, and strain of our mice based on past osteoporosis studies on Earth^55,102,103^. For age, 30 weeks is the approximate timepoint at which mouse hind limb bone mineral density (BMD) reaches its peak and stabilizes (i.e., skeletal maturity) until its eventual decline around two years of age^98,99^. Therefore, changes in BMD of 30-week-old mice, after 9 weeks of spaceflight, could be attributed more to the effects of microgravity than developmental processes. Prior spaceflight rodent studies have also solely evaluated young, skeletally-immature mice^47^ using animals that are 20-weeks-of-age or younger^44^. However, older, skeletally-mature mice (such as 30-week-old mice) display marked differences in bone physiology compared to younger animals, including a greater predisposition for bone homeostasis as opposed to bone formation and more significant microgravity-induced bone loss^47^. For sex, female mice are often favored over male mice in osteoporosis research as ovariectomy is the best-established, most clinically relevant model of postmenopausal osteoporosis^104^. Finally, regarding strain, BALB/c mice are commonly used for osteoporosis therapy testing because females respond optimally to ovariectomy and hypogonadism compared to other strains^45^ and males demonstrate more prevalent glucocorticoid-induced secondary osteoporosis than C57BL/6 males^46^. Yet, to our best knowledge, no previous studies have used BALB/c mice for microgravity-induced bone loss as these studies were primarily conducted using C57 animals instead^44^.

Methods were performed in accordance with relevant guidelines and regulations and approved by the Institutional Animal Care and Use Committees (IACUC) for flight at the Kennedy Space Center (Protocol# NAS-16-001-Y1), and the Chancellor’s Animal Research Committee at UCLA (Protocol #2009-127). The mice housed at UCLA were in pathogen-free ventilated cages, in a light and temperature-controlled environment, and were fed *ad libitum*. The mice in the ISS and the KSC were housed in NASA’s Rodent Research Hardware System and were fed a food bar diet *ad libitum*. The food bar consists of standard rodent chow in the shape of a bar, with moisture added to prevent floating and dissociation in microgravity. All mice in this study survived spaceflight and no obvious health issues were observed during the study. Necropsy was performed for each mouse at the terminal time point. Vital organs from each mouse were carefully dissected out, weighed, and photographed. No abnormality was observed between flight and ground control mice. Supplementary Table 1 shows the weights of each vital organ. No significant difference was found among all groups.

### Biodistribution study in vivo

We used the IVIS Lumina II Optical Imaging System to determine the bone targeting effect of BP-NELL-PEG in mice as previously described^42^. Eight 12-week-old female CD-1 mice (Taconic Biosciences, NY) were randomly assigned to the following groups: BP-NELL-PEG (1.25 mg kg^−1^ protein dose; 4 mice/group), PBS/PEG background control (the same amount of PEG as in BP-NELL-PEG, dissolved in PBS; 2 mice/group), and NELL-PEG (1.25 mg kg^−1^ protein dose; 2 mice/group). Based on our previous biodistribution testing, the standard deviation of the control group is much smaller than the treated group, so we reduced mice numbers in control groups to minimize animal distress based on the requirement of US animal welfare regulations (Animal Welfare Act, 7 U.S.C. 2131 et seq.). The injectate proteins were labeled with VivoTag 680XL and intravenously injected into mice *via* the lateral tail vein once (100 µl IV bolus dose) as previously described^42^. After 48 h post-injection, organs (femur, tibia, vertebrae, calvaria, liver, kidney, spleen, lung, brain, heart, muscle, ovary and fat) were collected and imaged by the IVIS ®Lumina II Optical Imaging System. The protein uptake amount was quantified by one gram of tissue weight.

### Osteogenicity study in ovariectomy-induced osteoporotic mice

Prior to the study of BP-NELL-PEG in spaceflight animals, we evaluated the osteogenic efficacy of BP-NELL-PEG in an ovariectomy (OVX)-induced osteoporotic mouse model using microCT measurements of trabecular BMD and BV/TV in weight-bearing bone. Forty 16-week-old female BALB/c mice (Taconic Biosciences, NY) were randomly assigned to the following groups (*n* = 8/group): Sham control, ovariectomy (OVX) control, OVX + BP-conjugated bovine serum albumin (BP-BSA), OVX + NELL-PEG, and OVX + BP-NELL-PEG. Osteoporosis was induced for 4 weeks post-OVX. Bone loss was confirmed by DXA (PIXImus 2, GE Lunar Corp., USA) pre- and post-OVX. Next, animals received intraperitoneal injections of BP-BSA (5.11 mg kg^−1^ dose; the same BP amount as BP-NELL-PEG), NELL-PEG (10 mg kg^−1^ protein dose) or BP-NELL-PEG (10 mg kg^−1^ protein dose) every 2 weeks. After 8 weeks of treatment, animals were euthanized and the distal femur, proximal tibia, and lumbar vertebrae were analyzed by microCT.

### Osteogenicity study in spaceflight mice – Logistics of the NASA Rodent Research (RR)-5 mission

The NASA Rodent Research (RR) series of missions began in 2014 to advance spaceflight research while embracing the advantages of using rodent models, which provide relevant insight and valuable extrapolations for predicting human physiological responses to long-duration spaceflight. Here we describe our spaceflight study, designated as RR-5, which extended spaceflight exposure of live mice to 60 days and utilized the maximum hardware capacity of 40 mice to assess bone loss patterns in long-duration spaceflight. Additionally, we utilized Live Animal Return (LAR), a specialized and coordinated process enabling transport of live animals from onboard the International Space Station (ISS) to a ground laboratory within 72 h, allowing quick access to flight-exposed animals for more expert analyses using ground capabilities not available on the ISS.

80 female BALB/c mice at 30-weeks of age were selected from 200 total animals based on body mass and DXA measurements to ensure skeletal maturity and baseline consistency. After being cleared of a final health check by the Kennedy Space Center (KSC) and NASA’s attending veterinarians, 40 mice were loaded into Rodent Transporters at L-26 h and turned over to SpaceX for installation in the Dragon capsule for launch, while the remaining 40 animals were housed at KSC as ground controls. Transportation from KSC to the ISS occurred during the SpaceX CRS-11 cargo resupply mission that launched on June 3^rd^, 2017. During flight, mice were allowed *ad libitum* access to water and specially developed NASA Nutrient Food Bars while daily real-time monitoring of physical health and behavior were conducted by KSC *via* live video feed, now stored in the NASA Ames Life Sciences Data Archive. Ground control mice at KSC were housed in identical hardware to flight mice, under matched environmental conditions (temperature, humidity, and carbon dioxide levels), and allowed *ad libitum* access to water and specially developed NASA Nutrient Food Bars. Starting at 1 week in orbit and every other week thereafter, flight mice received either PBS (*n* = 20 mice) or BP-NELL-PEG (*n* = 20 mice) injections. All flight treatment timepoints were exactly matched in the ground control groups. At 4.5 weeks post-launch, 20 of the 40 flight mice (*n* = 10 PBS treated and *n* = 10 BP-NELL-PEG treated) were returned to UCLA in the first use of NASA’s LAR system. Briefly, splash-down of the 20 mice occurred in the Pacific Ocean on July 3rd, 2017, where they were picked up *via* barge by SpaceX and transferred to NASA’s SeaVan for transport to Long Beach Pier, CA. There, NASA transported the live-return mice to UCLA’s research facilities for continued study. 20 ground control animals from KSC were also transported to UCLA at this time point *via* FedEx with temperature and humidity control. These 40 mice were transported to UCLA for continued study and designated the LAR group (data not reported here). The remaining 20 ISS flight mice (*n* = 10 PBS treated and *n* = 10 BP-NELL-PEG treated) and 20 KSC ground control mice (*n* = 10 PBS treated and *n* = 10 BP-NELL-PEG treated), designated the TERM group, remained until the terminal time point. At 9 weeks, all mice from LAR and TERM groups were euthanized and frozen, and TERM group samples were returned to UCLA. Frozen carcasses were thawed on wet ice before tissue dissection. Bones (femur, tibia and lumbar) were collected and fixed in 10% neutral formalin buffer for 6 days before subsequent analyses. We report here our findings for our TERM groups, which were exposed to the full 9 weeks of spaceflight.

### Osteogenicity study in spaceflight mice - DXA and MicroCT analyses

We used aBMD measurements from DXA to ascertain how BMD of whole bones - containing both cortical and trabecular compartments - were affected by microgravity and/or BP-NELL-PEG treatment. DXA images were taken for each mouse before launch, 4.5-week post-launch and 9-week post-launch, using a PIXImus2 mouse densitometer (GE Lunar Corp., USA) under anesthesia, following our previous standardized protocol^55,103^. The regions-of-interest for analysis were distal femur metaphysis, proximal tibial metaphysis and L6 lumbar vertebral body (Fig. 4a).

We also used MicroCT to evaluate the vBMD and microarchitecture of smaller volumes containing solely trabecular bone, in order to assess heterogenous site-specific changes and provide a more nuanced understanding of bone quality and subsequent fracture risk. For microCT analysis, femurs, tibias and lumbar vertebrae were scanned using Skyscan 1172 microCT (Bruker MicroCT N.V.) at an image resolution of 10 µm (55 kV and 181 mA radiation source, 0.5 mm aluminum filter). MicroCT reconstruction and analysis were performed as previously described^42^. 3D morphometric analyses of the distal femur, proximal tibia and L6 lumbar vertebral body were performed using CT-Analyzer software (SkyScan 1172, Belgium). The ROI for distal femur and proximal tibia was 1.0 mm of bone from the growth plate with a clearance of 0.3 mm from the growth plate to exclude the amount of natural growth. Thresholds were set at 90 for trabecular bone analysis and 150 for cortical bone analysis. The ROI for lumbar growth plate was individually specified, extending up from the caudal fused zygapophyses to include the entire caudal growth plate region. The threshold was set at 90. Morphometric parameters were then computed from the binarized images using direct 3D techniques (marching cubes and sphere-fitting methods), and included bone mineral density (BMD, g cm^−3^), percent bone volume (BV/TV, %), trabecular number (Tb.N, mm^−1^), trabecular thickness (Tb.Th, mm) and trabecular separation (Tb.Sp, mm).

### Osteogenicity study in spaceflight mice - Histology, TRAP staining and Immunohistochemical analysis

We assessed osteoblast and osteoclast activity in response to microgravity and BP-NELL-PEG treatment via semi-quantitation of staining for TRAP, osteocalcin, and RANKL in mouse femurs. Formalin-fixed paraffin-embedded sections of mouse bones (5 μm thick) from femur were cut with a microtome and mounted on charged slides (*n* = 3 per group). Sections were either subjected to standard hematoxylin-eosin (H&E) staining for overview or TRAP (Sigma-Aldrich, Cat# 387A-1KT) staining, and immunolabeled with the following antibodies: osteocalcin (Santa Cruz, Cat# sc-365797) and RANKL (Abcam, Cat# ab45039). Photomicrographs were acquired using Olympus BX 51 and IX 71 microscopes equipped with the Cell Sense digital imaging system (Olympus, Japan). To assess immunohistochemistry, digitized photographs were acquired and the intensity of positive staining was automatically measured using ImageJ 1.45 (National Institute of Health, freeware imaging software, https://imagej.nih.gov/nih-image/).

### Statistics

Statistics were performed in consultation with the UCLA Institute for Digital Research and Education, Statistical Computing Group. All experiments presented in figures are representative of at least three independent repetitions. Numerical data and histograms are expressed as the mean ± standard error of the mean or mean ± standard deviation. In comparisons between two groups, a two-tailed Student’s t-test was performed. In multiple group comparisons, one-way or two-way ANOVA was employed with Tukey’s method as a post-hoc adjustment.

To model the trajectory of DXA BMD over time, a linear mixed model was used, with a random intercept term to account for repeated measures within each mouse for each bone site analyzed. The fixed effects of the linear mixed model include four-way interaction of the factors: TIME, BP-NELL-PEG/PBS, FLIGHT/GC, LAR/TERM and all lower-order terms to allow for flexibility in modeling of effects. Linear mixed models were followed by likelihood ratio tests to assess (1) the cumulative contribution of all factors to the fit of the model and (2) the individual contribution of each factor (time, BP-NELL-PEG treatment, spaceflight) to the fit of the model (by testing model fit against a model where a factor is omitted). When a test for an individual factor was significant, pairwise comparisons were performed to test for specific effects of that factor across levels of the other factors. The mixed model direct results can be found in Supplementary Table 2. We want to reiterate that the contrasts reflecting specific hypotheses (e.g., do flight mice show a different % change in DXA compared to ground mice?) are the focus of the results section, not the parameters of the linear mixed model itself.

For lipid count analysis in histology, a linear mixed model was used with random intercept by mouse, and fixed effects of BP-NELL-PEG/PBS, flight/ground control, and their interactions. Pairwise contrasts were then performed to test for specific effects of BP-NELL-PEG/PBS and flight/ground control. All statistical analyses were performed with Stata 14 (StateCorp) software. Only *p*-values less than 0.05 were considered statistically significant.

### Reporting summary

Further information on research design is available in the Nature Research Reporting Summary linked to this article.

### Supplementary information

Supplemental Material

Reporting Summary

## Data Availability

All relevant data are included in the main text and supplementary materials. They are also available from the corresponding author upon reasonable request.
