# Supplementary material for: Bisphosphonate conjugation enhances the bone-specificity of NELL-1-based systemic therapy for spaceflight-induced bone loss in mice
Source: NPJ Microgravity. 2023 Sep 18;9:75. doi: 10.1038/s41526-023-00319-7 (PMC10507033; doi:10.1038/s41526-023-00319-7)
Supplement: Supplementary file 1 — Supplemental Material [file 41526_2023_319_MOESM1_ESM.pdf]

"Bisphosphonate conjugation enhances the bone-specificity of NELL-1-based systemic therapy for spaceflight-induced bone loss in mice"

Pin Ha[1,2,#], Jin Hee Kwak[3,#], Yulong Zhang[4,8,#], Jiayu Shi[9], Luan Tran[1,2,9], Timothy Pan Liu[1,2], Hsin-Chuan Pan[9], Samantha Lee[1,2], Jong Kil Kim[1], Eric Chen[9], Yasaman Shirazi-Fard[5], Louis S. Stodieck[6], Andy Lin[7], Zhong Zheng[1,2], Stella Nuo Dong[9], Xinli Zhang[9], Benjamin M. Wu[2,4,8*], Kang Ting[8*], Chia Soo[1,2,4*].

[1]Division of Plastic and Reconstructive Surgery, Department of Surgery, David Geffen School of Medicine, University of California, Los Angeles, Los Angeles, CA 90095; [2]Department of Orthopaedic Surgery and the Orthopaedic Hospital Research Center, David Geffen School of Medicine, University of California, Los Angeles, Los Angeles, CA 90095; [3]Herman Ostrow School of Dentistry, University of Southern California, Los Angeles, CA 90095; [4]Department of Bioengineering, School of Engineering, University of California, Los Angeles, Los Angeles, CA 90095; [5]Space Biosciences Division, NASA Ames Research Center, Moffett Field, CA 94035; [6]BioServe Space Technologies and Aerospace Engineering Sciences, University of Colorado, Boulder, CO 80303; [7]Office of Advanced Research Computing, University of California, Los Angeles, Los Angeles, CA 90095; [8]Forsyth Institute, Cambridge, MA 02142; [9]School of Dentistry, University of California, Los Angeles, Los Angeles, CA 90095.

[#] These authors contributed to the work equally and should be regarded as co-first authors.

* Co-corresponding authors:

Benjamin M. Wu: bwu@forsyth.org

Kang Ting: erickangting@gmail.com

Chia Soo: bsoo@ucla.edu

Supplementary Tables

Supplementary Table 1. Vital organ weights of study mice

Weight (g)	Ground + PBs		Flight + PBS		Ground + BP-NELL-PEG		Flight + BP-NELL-PEG	
	Mean	SD	Mean	SD	Mean	SD	Mean	SD
**liver**	0.945	0.060	0.964	0.279	0.987	0.357	0.963	0.357
**pancreas**	0.210	0.278	0.211	0.053	0.232	0.071	0.218	0.071
**spleen**	0.102	0.304	0.093	0.072	0.111	0.085	0.103	0.085
**stomach**	0.192	0.288	0.215	0.102	0.193	0.103	0.217	0.103
**heart**	0.177	0.016	0.160	0.080	0.142	0.079	0.135	0.079
**kidney**	0.269	0.324	0.265	0.073	0.260	0.080	0.266	0.080
**lung**	0.227	0.272	0.229	0.082	0.189	0.077	0.202	0.077

Supplementary Table 2. Direct results of linear mixed model analysis on DXA data

Term	Estimate	Std. Error	df	t-value	Pr(>|t|)	
(Intercept)	0.2137	0.0186	124.1748	11.49	2.00E-16	***
Time	-0.0112	0.0185	76.4375	-0.6	0.54803	
Treatment/PBS	0.011	0.0263	124.1748	0.42	0.67723	
Ground/Flight	-0.0961	0.0269	128.133	-3.58	0.00049	***
LAR/TERM	-0.1803	0.0263	124.1748	-6.85	3.00E-10	***
Time : Treatment/PBS	0.0593	0.0262	76.4375	2.27	0.02621	*
Time : Ground/Flight	0.0889	0.0267	77.9748	3.33	0.00133	**
Treatment/PBS : Ground/Flight	0.0716	0.0381	126.1511	1.88	0.06257	.
Time : LAR/TERM	0.0382	0.0262	76.4375	1.46	0.14784	
Treatment/PBS : LAR/TERM	0.0394	0.0381	128.5995	1.04	0.30246	
Ground/Flight : LAR/TERM	-0.0214	0.0376	126.2046	-0.57	0.57007	
Time : Treatment/PBS : Ground/Flight	-0.0962	0.0379	77.1973	-2.54	0.01312	*
Time : Treatment/PBS : LAR/TERM	-0.0533	0.0379	78.1604	-1.41	0.1633	
Time : Ground/Flight : LAR/TERM	-0.0865	0.0374	77.2181	-2.31	0.02335	*
Treatment/PBS : Ground/Flight : LAR/TERM	-0.0255	0.0539	127.3789	-0.47	0.63629	
Time : Treatment/PBS : Ground/Flight : LAR/TERM	0.0629	0.0535	77.677	1.17	0.24374	

Supplementary Figures

Supplementary Figure 1.

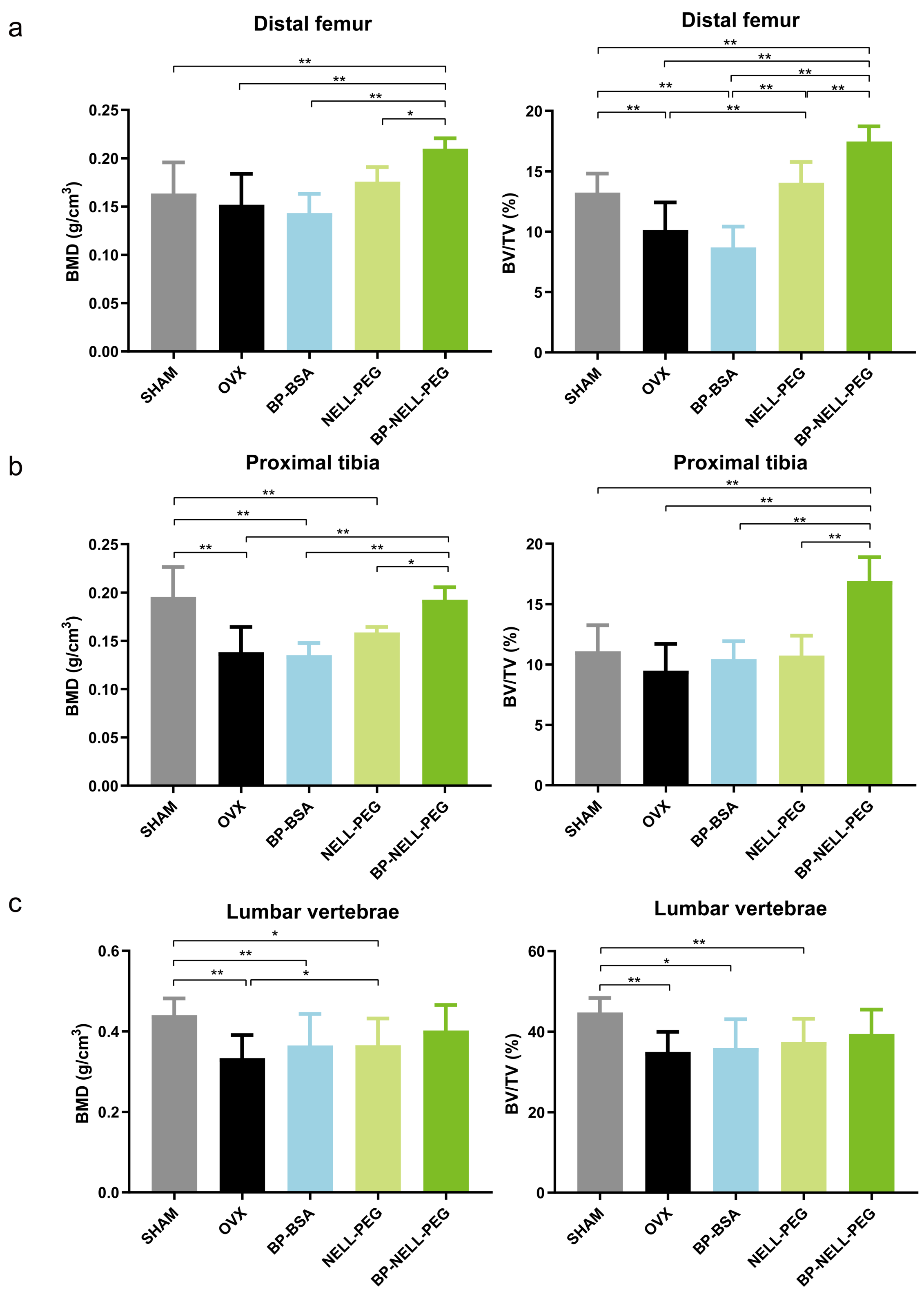

Supplemental Figure 1. Osteogenicity study in ovariectomy-induced osteoporotic mice and verification of BP's absence of OC activity. BP-BSA carrier control, NELL-PEG, and BP-NELL-PEG were intraperitoneally injected every-2-weeks (dose: 10 mg/kg NELL-1 protein) in ovariectomy (OVX)-induced osteoporotic mice, with OVX controls and Sham (healthy) controls receiving intraperitoneal PBS. After 8 weeks of injection, distal femurs, proximal tibia and lumbar vertebrae were analyzed with microCT. BP-NELL-PEG significantly enhanced trabecular BMD and BV/TV in the **(a)** femurs, **(b)** tibias, and **(c)** lumbar vertebrae of osteoporotic mice to a level comparable to /or higher than Sham control. Importantly, BP-BSA carrier control showed no osteogenic potential and verified that the BP conjugate retained no anti-osteoclastic activity *in vivo.* Data are presented as means ± SD. n = 8 per group. *$p<0.05$, **$p<0.01$.

Supplementary Figure 2.

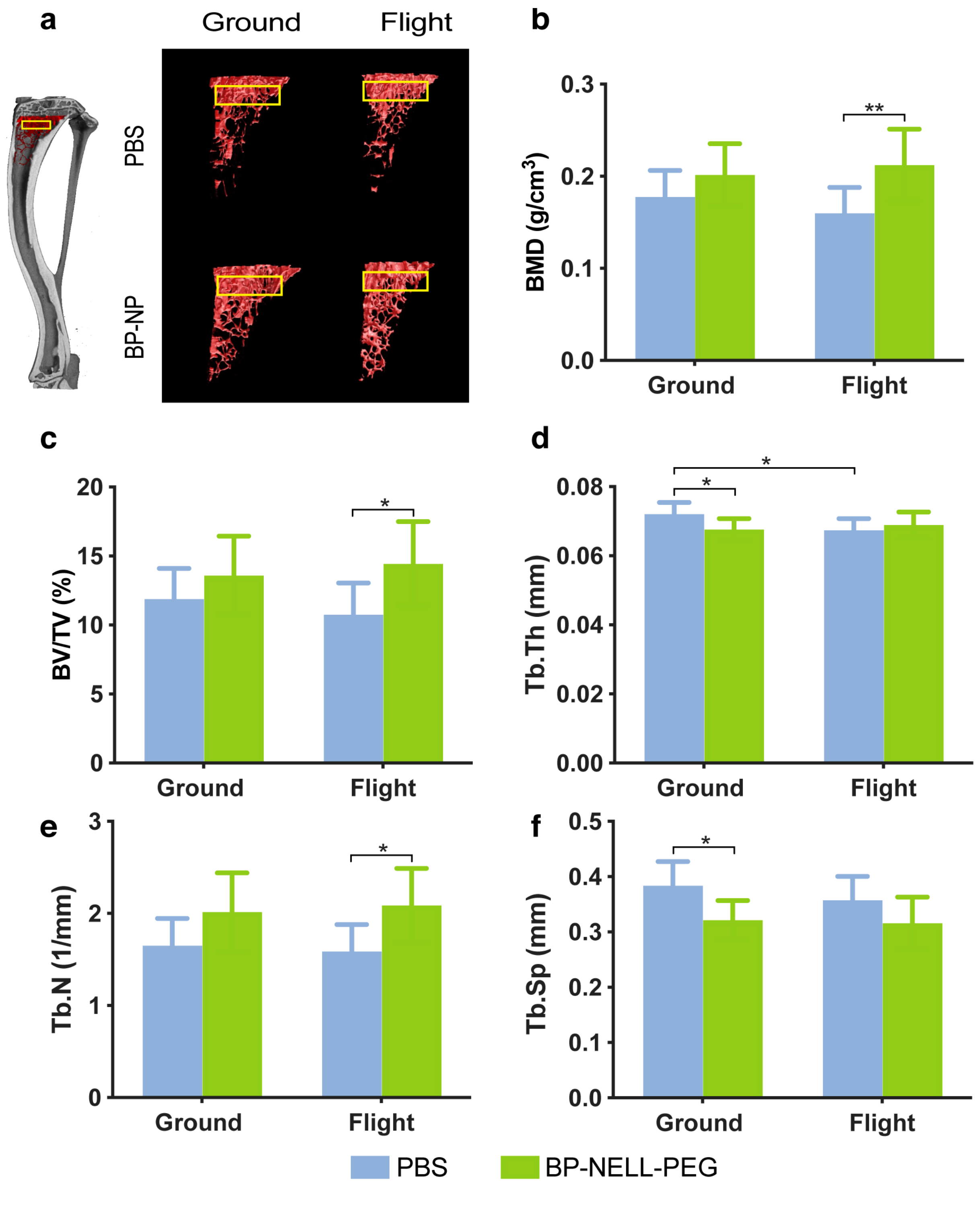

Supplemental Figure 2. MicroCT analysis of tibiae after 9 weeks of spaceflight. (a) Representative microCT 3D reconstruction images of the proximal tibial metaphyseal region (ROI within the trabecular bone labeled in yellow boxes). (b-f) Histograms represent the trabecular structural parameters of the proximal tibiae: bone mineral density (BMD), percent bone volume (BV/TV), trabecular thickness (Tb.Th), trabecular number (Tb.N), and trabecular separation (Tb.Sp). Data are presented as means ± SD. n = 10 per group. $^*p<0.05$, $^{**}p<0.01$.

Supplementary Figure 3.

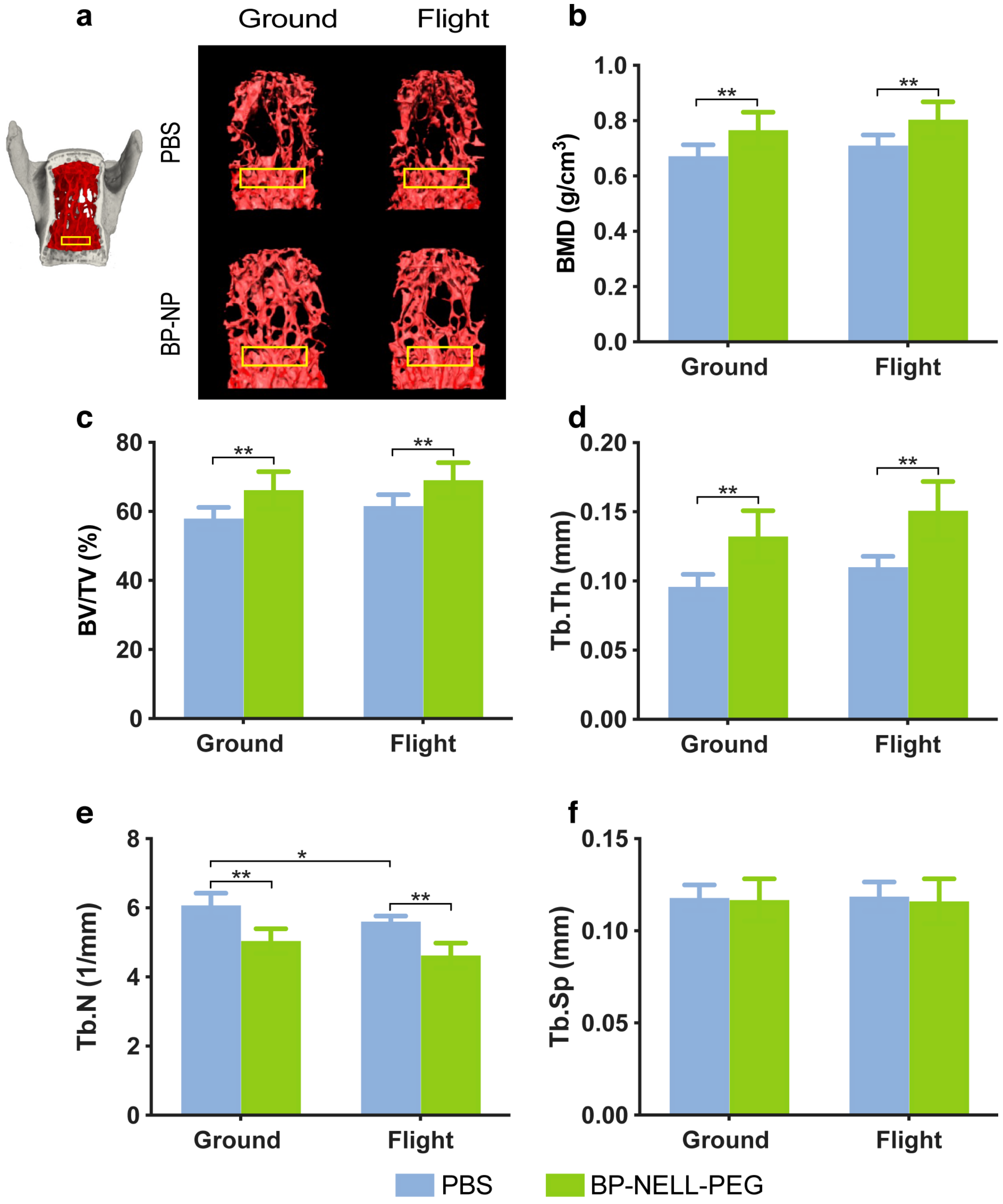

Supplementary Figure 3. MicroCT analysis of lumbar vertebrae after 9 weeks of spaceflight. (a) Representative microCT 3D reconstruction images of the lumbar vertebral metaphyseal region (ROI within the trabecular bone was labeled in yellow boxes). (b-f) Histograms represent the trabecular structural parameters of the lumbar vertebrae: bone mineral density (BMD), percent bone volume (BV/TV), trabecular thickness (Tb.Th), trabecular number (Tb.N), and trabecular separation (Tb.Sp). Data are presented as means ± SD. n = 10 per group. *$p<0.05$, **$p<0.01$.

Supplementary Figure 4.

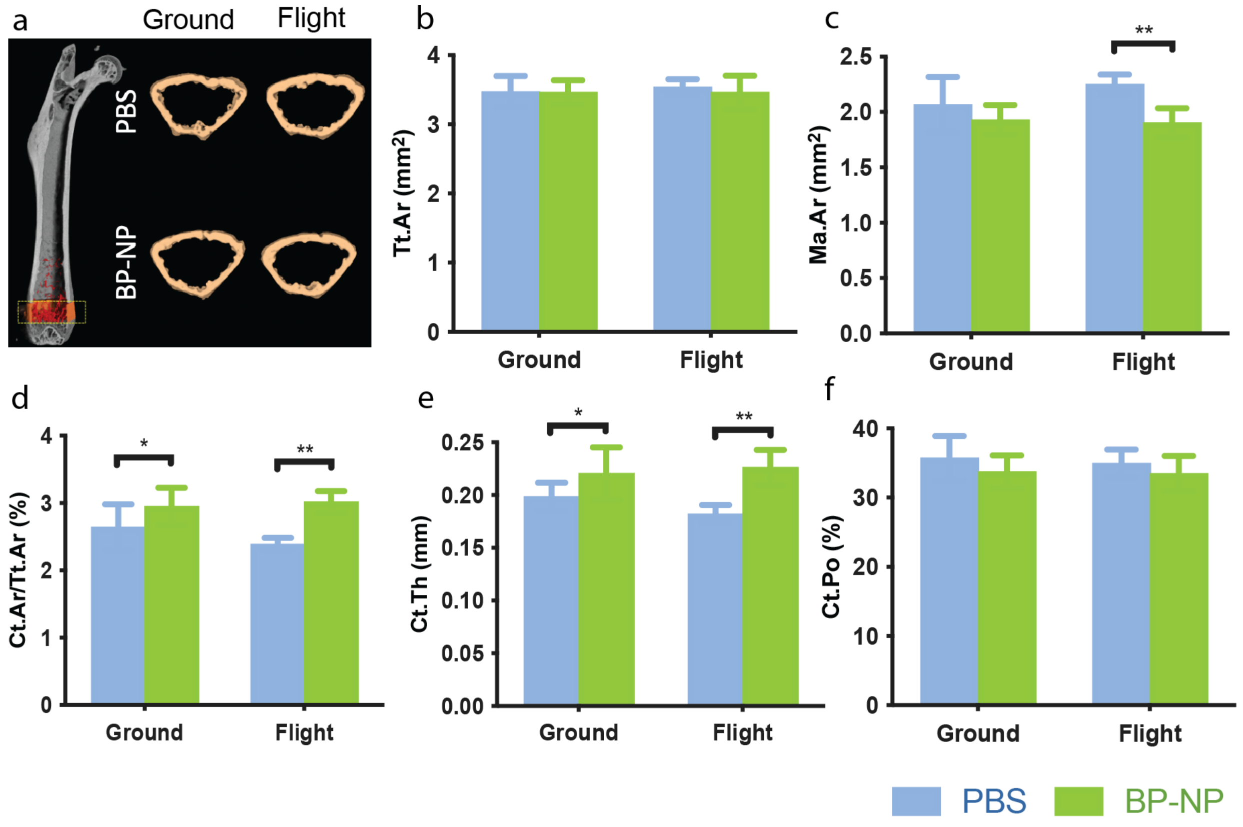

Supplementary Figure 4. Cortical bone measurements from the distal femur. (a) Representative microCT regions of interest of cortical bone within the distal femur shown in orange. (b-f) Bar graphs represent the cortical structural parameters of the distal femur: total cross-sectional area inside periosteal envelope (Tt.Ar), medullary area (Ma.Ar), cortical area fraction (Ct.Ar/Tt.Ar), cortical thickness (Ct.Th), and cortical porosity (Ct.Po). Data are presented as means ± SD. n = 10 per group. *p<0.05, **p<0.01.
