# Supplementary material for: Bisphosphonate conjugation enhances the bone-specificity of NELL-1-based systemic therapy for spaceflight-induced bone loss in mice
Source: NPJ Microgravity. 2023 Sep 18;9:75. doi: 10.1038/s41526-023-00319-7 (PMC10507033; doi:10.1038/s41526-023-00319-7)
Supplement: Supplementary file 2 — Reporting Summary [file 41526_2023_319_MOESM2_ESM.pdf]

nature portfolio

Corresponding author(s): Chia Soo, Benjamin M. Wu, Kang Ting

Last updated by author(s): Jul 31, 2023



# Reporting Summary

Nature Portfolio wishes to improve the reproducibility of the work that we publish. This form provides structure for consistency and transparency in reporting. For further information on Nature Portfolio policies, see our Editorial Policies and the Editorial Policy Checklist.

## Statistics

For all statistical analyses, confirm that the following items are present in the figure legend, table legend, main text, or Methods section.

| n/a | Confirmed | |
|---|---|---|
| ☐ | ☒ | The exact sample size (*n*) for each experimental group/condition, given as a discrete number and unit of measurement |
| ☐ | ☒ | A statement on whether measurements were taken from distinct samples or whether the same sample was measured repeatedly |
| ☐ | ☒ | The statistical test(s) used AND whether they are one- or two-sided<br>*Only common tests should be described solely by name; describe more complex techniques in the Methods section.* |
| ☒ | ☐ | A description of all covariates tested |
| ☐ | ☒ | A description of any assumptions or corrections, such as tests of normality and adjustment for multiple comparisons |
| ☐ | ☒ | A full description of the statistical parameters including central tendency (e.g. means) or other basic estimates (e.g. regression coefficient) AND variation (e.g. standard deviation) or associated estimates of uncertainty (e.g. confidence intervals) |
| ☒ | ☐ | For null hypothesis testing, the test statistic (e.g. *F*, *t*, *r*) with confidence intervals, effect sizes, degrees of freedom and *P* value noted<br>*Give P values as exact values whenever suitable.* |
| ☒ | ☐ | For Bayesian analysis, information on the choice of priors and Markov chain Monte Carlo settings |
| ☒ | ☐ | For hierarchical and complex designs, identification of the appropriate level for tests and full reporting of outcomes |
| ☒ | ☐ | Estimates of effect sizes (e.g. Cohen's *d*, Pearson's *r*), indicating how they were calculated |

*Our web collection on statistics for biologists contains articles on many of the points above.*

## Software and code

Policy information about availability of computer code

| | |
|---|---|
| Data collection | 1. DXA data were collected by Lunar Piximus software; microCT data were collected by Skyscan 1172 (Bruker company).<br>2. Staining photos were captured by Olympus CellSens standard-microscope imaging software. |
| Data analysis | 1. The DXA parameters were analyzed by Lunar Piximus software; microCT 3D reconstruction images were constructed by NRecon, DataViwer, and CT Analyser software.<br>2. Staining photos were captured by Olympus CellSens standard-microscope imaging software.<br>3. Graphpad prism 8 (Graphpad Software, La Jolla, CA) was used to analyze the data.<br>4. Statistical analyses were performed with Stata 14 (StateCorp) software. |

For manuscripts utilizing custom algorithms or software that are central to the research but not yet described in published literature, software must be made available to editors and reviewers. We strongly encourage code deposition in a community repository (e.g. GitHub). See the Nature Portfolio guidelines for submitting code & software for further information.

# Data

Policy information about availability of data

All manuscripts must include a data availability statement. This statement should provide the following information, where applicable:

- Accession codes, unique identifiers, or web links for publicly available datasets
- A description of any restrictions on data availability
- For clinical datasets or third party data, please ensure that the statement adheres to our policy

All relevant data are included in the main text and supplementary materials. They are also available from the corresponding author upon reasonable request.

# Research involving human participants, their data, or biological material

Policy information about studies with human participants or human data. See also policy information about sex, gender (identity/presentation), and sexual orientation and race, ethnicity and racism.

| | |
|---|---|
| Reporting on sex and gender | N/A |
| Reporting on race, ethnicity, or other socially relevant groupings | N/A |
| Population characteristics | N/A |
| Recruitment | N/A |
| Ethics oversight | N/A |

Note that full information on the approval of the study protocol must also be provided in the manuscript.

# Field-specific reporting

Please select the one below that is the best fit for your research. If you are not sure, read the appropriate sections before making your selection.

☒ Life sciences ☐ Behavioural & social sciences ☐ Ecological, evolutionary & environmental sciences

For a reference copy of the document with all sections, see nature.com/documents/nr-reporting-summary-flat.pdf

# Life sciences study design

All studies must disclose on these points even when the disclosure is negative.

| | |
|---|---|
| Sample size | Unless otherwise specified, we chose the sample size, mouse age, gender, and strain based on past osteoporosis studies on Earth. |
| Data exclusions | No data were excluded from the analysis. |
| Replication | We only have access to this flight once. This is a spaceflight study which experiments cannot be replicated. |
| Randomization | The mice were randomly allocated into experimental groups to ensure there was no significant difference among mice in body weight. |
| Blinding | The investigators were blinded during data collection and analysis. |

# Reporting for specific materials, systems and methods

We require information from authors about some types of materials, experimental systems and methods used in many studies. Here, indicate whether each material, system or method listed is relevant to your study. If you are not sure if a list item applies to your research, read the appropriate section before selecting a response.

## Materials & experimental systems

| n/a | Involved in the study |
| --- | --- |
| ☐ | ☒ Antibodies |
| ☐ | ☒ Eukaryotic cell lines |
| ☒ | ☐ Palaeontology and archaeology |
| ☐ | ☒ Animals and other organisms |
| ☒ | ☐ Clinical data |
| ☒ | ☐ Dual use research of concern |
| ☒ | ☐ Plants |

## Methods

| n/a | Involved in the study |
| --- | --- |
| ☒ | ☐ ChIP-seq |
| ☒ | ☐ Flow cytometry |
| ☒ | ☐ MRI-based neuroimaging |

# Antibodies

| | |
| --- | --- |
| Antibodies used | anti-RANKL antibody (Abcam, ab216484); anti-OCN antibody (Santa Cruz Biotechnology, sc-365797) |
| Validation | The antibodies were validated by the manufacturer as per their websites. |

# Eukaryotic cell lines

Policy information about cell lines and Sex and Gender in Research

| | |
| --- | --- |
| Cell line source(s) | MC3T3 (Subclone 4, ATCC, CRL-2593) |
| Authentication | The cell line was validated by the manufacturer as per its website. |
| Mycoplasma contamination | Not detected based on the manufacturer as per its website. |
| Commonly misidentified lines (See ICLAC register) | N/A |

# Animals and other research organisms

Policy information about studies involving animals; ARRIVE guidelines recommended for reporting animal research, and Sex and Gender in Research

| | |
| --- | --- |
| Laboratory animals | Female BALB/c mice (Taconic Biosciences, NY) |
| Wild animals | N/A |
| Reporting on sex | Female mice were used in this study, because females are often favored over male mice in osteoporosis research as ovariectomy is the best-established, most clinically relevant model of post-menopausal osteoporosis. |
| Field-collected samples | N/A |
| Ethics oversight | Methods were performed in accordance with relevant guidelines and regulations and approved by the Institutional Animal Care and Use Committees (IACUC) for flight at the Kennedy Space Center (Protocol# NAS-16-001-Y1), and the Chancellor's Animal Research Committee at UCLA (Protocol #2009-127). |

Note that full information on the approval of the study protocol must also be provided in the manuscript.
